# 3,3,5,5-Tetra­methyl-*r*-2,*c*-6-diphenyl­piperidin-4-one

**DOI:** 10.1107/S1600536812018983

**Published:** 2012-06-13

**Authors:** C. Govindaraju, R. Valliappan, V. Sundari

**Affiliations:** a Department of Chemistry, Annamalai University, Annamalai Nagar 608 002, Tamil Nadu, India

## Abstract

The piperidone ring of the title compound, C_21_H_25_NO, adopts a chair conformation with the two phenyl groups equatorially oriented and *cis* to each other. In the crystal, mol­ecules are linked by weak N—H⋯O hydrogen bonds, forming chains parallel to [100].

## Related literature

For some bioactive properties of piperidones, see: Mobio *et al.* (1989 ▶). For piperidone ring conformations in related compounds, see: Parthiban *et al.* (2008 ▶); Lakshminarayana *et al.* (2009 ▶); Ravichandran *et al.* (2010 ▶). For the synthesis, see: Noller & Baliah (1948 ▶). For ring puckering parameters, see: Nardelli (1983 ▶); Cremer & Pople (1975 ▶).
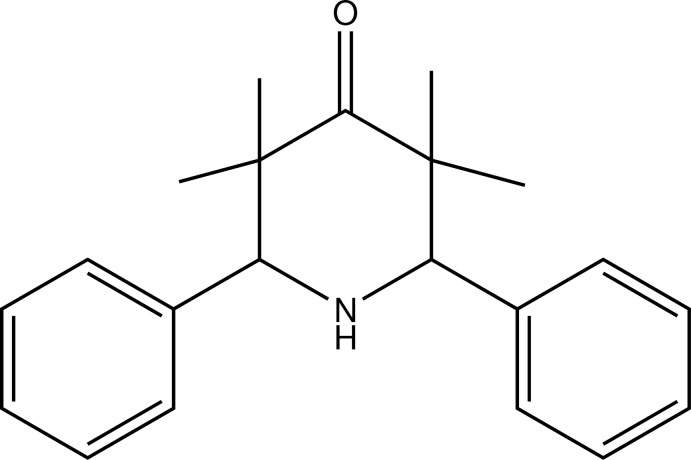


## Experimental

### 

#### Crystal data

C_21_H_25_NO
*M* *_r_* = 307.42
Triclinic, 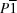

*a* = 6.9227 (11) Å
*b* = 11.540 (2) Å
*c* = 12.472 (2) Å
α = 64.771 (4)°
β = 80.755 (5)°
γ = 72.675 (4)°
*V* = 859.8 (3) Å^3^
*Z* = 2
Mo *K*α radiation
μ = 0.07 mm^−1^
*T* = 295 K
0.30 × 0.25 × 0.20 mm

#### Data collection

Bruker Kappa APEXII CCD diffractometer
Absorption correction: multi-scan (*SADABS*; Bruker, 2004 ▶) *T*_min_ = 0.919, *T*_max_ = 0.986
12752 measured reflections
2659 independent reflections
2123 reflections with *I* > 2σ(*I*)
*R*_int_ = 0.032
θ_max_ = 24.0°

#### Refinement

*R*[*F* ^2^ > 2σ(*F* ^2^)] = 0.056
*wR*(*F* ^2^) = 0.152
*S* = 1.15
2659 reflections
217 parameters
H atoms treated by a mixture of independent and constrained refinement
Δρ_max_ = 0.23 e Å^−3^
Δρ_min_ = −0.18 e Å^−3^

### 

Data collection: *APEX2* (Bruker, 2004 ▶); cell refinement: *APEX2* and *SAINT-Plus* (Bruker, 2004 ▶); data reduction: *SAINT-Plus* and *XPREP* (Bruker, 2004 ▶); program(s) used to solve structure: *SIR92* (Altomare *et al.*, 1993 ▶); program(s) used to refine structure: *SHELXL97* (Sheldrick, 2008 ▶); molecular graphics: *ORTEP-3* (Farrugia, 1997 ▶) and *Mercury* (Macrae *et al.*, 2008 ▶); software used to prepare material for publication: *PLATON* (Spek, 2009 ▶).

## Supplementary Material

Crystal structure: contains datablock(s) global, I. DOI: 10.1107/S1600536812018983/lr2058sup1.cif

Structure factors: contains datablock(s) I. DOI: 10.1107/S1600536812018983/lr2058Isup2.hkl

Supplementary material file. DOI: 10.1107/S1600536812018983/lr2058Isup3.cml

Additional supplementary materials:  crystallographic information; 3D view; checkCIF report

## Figures and Tables

**Table 1:** Hydrogen-bond geometry (Å, °)

*D*—H⋯*A*	*D*—H	H⋯*A*	*D*⋯*A*	*D*—H⋯*A*
N1—H1*A*⋯O1^i^	0.94 (3)	2.39 (3)	3.258 (3)	153 (2)

Symmetry code: (i) 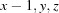.

**Fig. 1.**
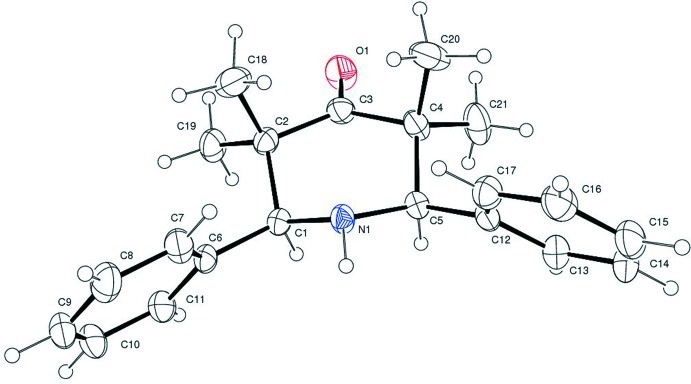
The molecular structure of the title compound. Displacement ellipsoids are drawn at the 40% probability level.

**Fig. 2.**
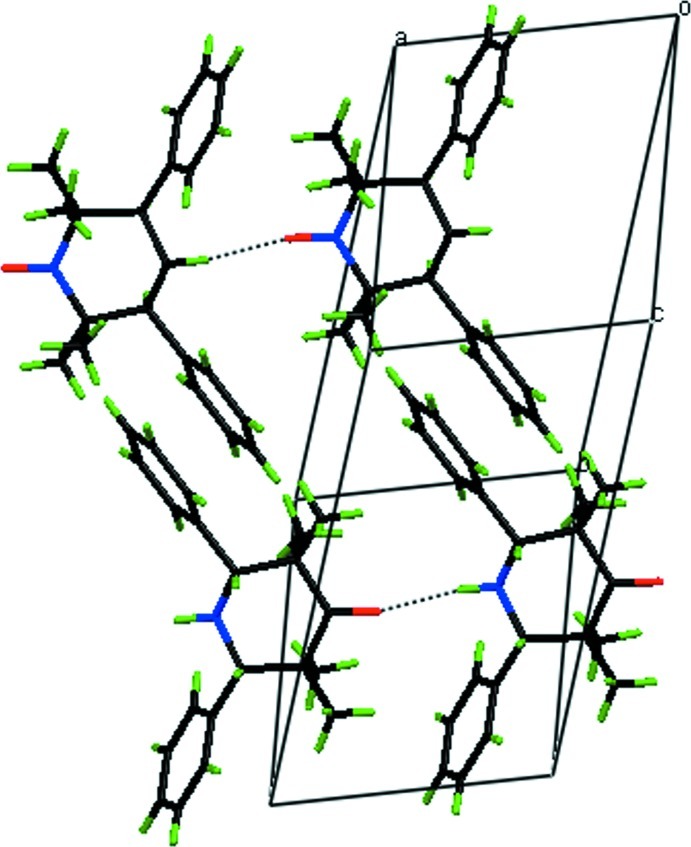
Part of crystal structure showing the packing of the molecules in the unit cell and formation of N1—H1A···O1 hydrogen bond.

## supplementary crystallographic information

### Comment

Aryl substituted piperidine-4-ones are important heterocyclic entities present in natural products like alkaloids.The piperidones exhibit diverse bioactivities such as bactericidal, fungicidal and herbicidal activities (Mobio *et al.*, 1989).The six-membered ring of piperidones adopts, predominantly, a chair conformation (Parthiban *et al.*, 2008) but many often the conformation depends on the substitution in the piperidone ring (Lakshminarayana *et al.*, 2009; Ravichandran *et al.*, 2010). The determination of the crystal structure of the title compound was mainly undertaken to evaluate the impact of four methyl groups at C2 and C4 carbon atoms. The *ORTEP* diagram of the title compound is shown in Fig. 1. In the title compound C_21_H_25_NO, the piperidone ring adopts a chair conformation with ring puckering parameters (Nardelli, 1983; Cremer & Pople, 1975) of Q=0.543 (2)°, θ = 155.55 (3)° and φ=136.72 (2)°. The two phenyl groups are equatorial oriented and *cis* to each other. The crystal packing is stabilized by a week N—H···O hydrogen bond [*d*(N—O)=3.257 (4) Å and angle N—H···O= 151.11 (2)°]. The N—H···O hydrogen bond connects the adjacent molecule into a head to tail fashion to generate a one dimensional chain extending parallel to the [1 0 0] direction.

### Experimental

The title compound was prepared by one pot synthesis from 3,4-dimethy-3-pentanone, benzaldehyde and ammonium acetate at 1:2:1 proportion in ethanol as solvent by adopting the literature procedure reported by (Noller & Baliah, 1948). Crystals suitable for single-crystal X-raydiffraction were grown by slow evaporation of a solution in benzene (m.p. 467–469 K).

### Refinement

The H atoms associated with the carbon atoms were fixed geometrically and allowed to ride on their parent carbon atoms with C–H distances in the range of 0.93 Å–0.98 Å and *U*_iso_(H) set to either 1.2*U*_equ_(C) or 1.5*U*_eqi_(C) of the carrier atom. The H-atom bound to the N-atom is identified from a difference electron density map and restrained to a distance of 0.93 (3) Å

### Crystal data

**Table:**

C_21_H_25_NO	*Z* = 2
*M**_r_* = 307.42	*F*(000) = 332
Triclinic, *P*1	*D*_x_ = 1.187 Mg m^−^^3^
Hall symbol: -P 1	Mo *K*α radiation, λ = 0.71073 Å
*a* = 6.9227 (11) Å	Cell parameters from 4779 reflections
*b* = 11.540 (2) Å	θ = 2.1–23.8°
*c* = 12.472 (2) Å	µ = 0.07 mm^−^^1^
α = 64.771 (4)°	*T* = 295 K
β = 80.755 (5)°	Block, colourless
γ = 72.675 (4)°	0.30 × 0.25 × 0.20 mm
*V* = 859.8 (3) Å^3^	

### Data collection

**Table:**

Bruker Kappa APEXII CCD diffractometer	2659 independent reflections
Radiation source: fine-focus sealed tube	2123 reflections with *I* > 2σ(*I*)
Graphite monochromator	*R*_int_ = 0.032
ω and φ scan	θ_max_ = 24.0°, θ_min_ = 2.1°
Absorption correction: multi-scan (*SADABS*; Bruker, 2004)	*h* = −7→7
*T*_min_ = 0.919, *T*_max_ = 0.986	*k* = −13→13
12752 measured reflections	*l* = −14→14

### Refinement

**Table:**

Refinement on *F*^2^	Secondary atom site location: difference Fourier map
Least-squares matrix: full	Hydrogen site location: inferred from neighbouring sites
*R*[*F*^2^ > 2σ(*F*^2^)] = 0.056	H atoms treated by a mixture of independent and constrained refinement
*wR*(*F*^2^) = 0.152	*w* = 1/[σ^2^(*F*_o_^2^) + (0.0562*P*)^2^ + 0.4957*P*] where *P* = (*F*_o_^2^ + 2*F*_c_^2^)/3
*S* = 1.15	(Δ/σ)_max_ < 0.001
2659 reflections	Δρ_max_ = 0.23 e Å^−^^3^
217 parameters	Δρ_min_ = −0.18 e Å^−^^3^
0 restraints	Extinction correction: *SHELXL97* (Sheldrick, 2008), Fc^*^=kFc[1+0.001xFc^2^λ^3^/sin(2θ)]^-1/4^
Primary atom site location: structure-invariant direct methods	Extinction coefficient: 0.020 (4)

### Special details

**Table:**

Geometry. All e.s.d.'s (except the e.s.d. in the dihedral angle between two l.s. planes) are estimated using the full covariance matrix. The cell e.s.d.'s are taken into account individually in the estimation of e.s.d.'s in distances, angles and torsion angles; correlations between e.s.d.'s in cell parameters are only used when they are defined by crystal symmetry. An approximate (isotropic) treatment of cell e.s.d.'s is used for estimating e.s.d.'s involving l.s. planes.
Refinement. Refinement of *F*^2^ against ALL reflections. The weighted *R*-factor *wR* and goodness of fit *S* are based on *F*^2^, conventional *R*-factors *R* are based on *F*, with *F* set to zero for negative *F*^2^. The threshold expression of *F*^2^ > σ(*F*^2^) is used only for calculating *R*-factors(gt) *etc*. and is not relevant to the choice of reflections for refinement. *R*-factors based on *F*^2^ are statistically about twice as large as those based on *F*, and *R*- factors based on ALL data will be even larger.

### Fractional atomic coordinates and isotropic or equivalent isotropic displacement parameters (Å^2^)

**Table:**

	*x*	*y*	*z*	*U*_iso_*/*U*_eq_	
C1	0.7201 (4)	0.4000 (2)	0.1945 (2)	0.0372 (6)	
H1	0.7165	0.4280	0.1087	0.045*	
C2	0.9321 (4)	0.3963 (2)	0.2220 (2)	0.0407 (6)	
C3	1.0898 (4)	0.2903 (2)	0.1901 (2)	0.0435 (6)	
C4	1.0442 (4)	0.1603 (2)	0.2102 (2)	0.0446 (6)	
C5	0.8169 (4)	0.1810 (2)	0.1951 (2)	0.0386 (6)	
H5	0.7920	0.2263	0.1100	0.046*	
C6	0.5498 (4)	0.4960 (2)	0.2302 (2)	0.0394 (6)	
C7	0.4653 (4)	0.4602 (3)	0.3438 (2)	0.0496 (7)	
H7	0.5133	0.3750	0.4003	0.059*	
C8	0.3102 (5)	0.5492 (3)	0.3749 (3)	0.0633 (8)	
H8	0.2544	0.5234	0.4518	0.076*	
C9	0.2383 (5)	0.6751 (3)	0.2932 (3)	0.0657 (9)	
H9	0.1351	0.7354	0.3144	0.079*	
C10	0.3196 (5)	0.7116 (3)	0.1799 (3)	0.0641 (9)	
H10	0.2705	0.7970	0.1239	0.077*	
C11	0.4734 (4)	0.6232 (2)	0.1478 (3)	0.0514 (7)	
H11	0.5265	0.6493	0.0703	0.062*	
C12	0.7552 (4)	0.0522 (2)	0.2417 (2)	0.0408 (6)	
C13	0.7551 (4)	−0.0084 (3)	0.1664 (3)	0.0533 (7)	
H13	0.7920	0.0311	0.0866	0.064*	
C14	0.7008 (5)	−0.1267 (3)	0.2086 (3)	0.0647 (9)	
H14	0.7004	−0.1655	0.1568	0.078*	
C15	0.6482 (5)	−0.1867 (3)	0.3250 (3)	0.0655 (9)	
H15	0.6137	−0.2669	0.3532	0.079*	
C16	0.6461 (4)	−0.1284 (3)	0.4009 (3)	0.0597 (8)	
H16	0.6098	−0.1689	0.4807	0.072*	
C17	0.6982 (4)	−0.0093 (2)	0.3588 (2)	0.0483 (7)	
H17	0.6945	0.0300	0.4108	0.058*	
C18	0.9507 (5)	0.3605 (3)	0.3540 (3)	0.0632 (8)	
H18A	0.9024	0.2832	0.4010	0.095*	
H18B	0.8713	0.4332	0.3736	0.095*	
H18C	1.0900	0.3429	0.3700	0.095*	
C19	0.9807 (4)	0.5297 (3)	0.1504 (3)	0.0569 (8)	
H19A	1.1159	0.5236	0.1653	0.085*	
H19B	0.8867	0.5960	0.1733	0.085*	
H19C	0.9702	0.5535	0.0675	0.085*	
C20	1.1125 (5)	0.0629 (3)	0.3352 (3)	0.0691 (9)	
H20A	1.2464	0.0646	0.3447	0.104*	
H20B	1.1131	−0.0249	0.3467	0.104*	
H20C	1.0207	0.0879	0.3927	0.104*	
C21	1.1721 (5)	0.1079 (3)	0.1211 (3)	0.0707 (10)	
H21A	1.1335	0.1695	0.0422	0.106*	
H21B	1.1507	0.0238	0.1359	0.106*	
H21C	1.3126	0.0973	0.1293	0.106*	
N1	0.6901 (3)	0.26610 (18)	0.25244 (18)	0.0388 (5)	
O1	1.2574 (3)	0.30563 (19)	0.1531 (2)	0.0641 (6)	
H1A	0.554 (4)	0.268 (2)	0.251 (2)	0.042 (7)*	

### Atomic displacement parameters (Å^2^)

**Table:**

	*U*^11^	*U*^22^	*U*^33^	*U*^12^	*U*^13^	*U*^23^
C1	0.0395 (14)	0.0324 (12)	0.0436 (14)	−0.0100 (10)	−0.0031 (10)	−0.0179 (11)
C2	0.0389 (14)	0.0400 (14)	0.0502 (15)	−0.0132 (11)	−0.0018 (11)	−0.0225 (12)
C3	0.0344 (15)	0.0428 (14)	0.0531 (15)	−0.0111 (11)	−0.0032 (11)	−0.0179 (12)
C4	0.0370 (15)	0.0330 (13)	0.0583 (16)	−0.0050 (10)	0.0008 (11)	−0.0169 (12)
C5	0.0393 (14)	0.0320 (12)	0.0441 (14)	−0.0047 (10)	−0.0036 (10)	−0.0171 (11)
C6	0.0391 (14)	0.0319 (13)	0.0546 (16)	−0.0104 (10)	−0.0028 (11)	−0.0230 (12)
C7	0.0482 (16)	0.0434 (15)	0.0598 (17)	−0.0111 (12)	0.0030 (13)	−0.0254 (13)
C8	0.0566 (19)	0.073 (2)	0.078 (2)	−0.0182 (16)	0.0122 (15)	−0.0510 (18)
C9	0.0520 (19)	0.0570 (19)	0.105 (3)	−0.0006 (14)	−0.0078 (17)	−0.055 (2)
C10	0.063 (2)	0.0361 (15)	0.094 (2)	0.0003 (13)	−0.0180 (18)	−0.0303 (16)
C11	0.0548 (17)	0.0365 (14)	0.0644 (18)	−0.0099 (12)	−0.0086 (13)	−0.0207 (13)
C12	0.0366 (14)	0.0295 (12)	0.0541 (16)	−0.0022 (10)	−0.0059 (11)	−0.0176 (11)
C13	0.0601 (19)	0.0417 (15)	0.0627 (18)	−0.0091 (13)	−0.0059 (14)	−0.0266 (14)
C14	0.070 (2)	0.0442 (17)	0.093 (2)	−0.0083 (14)	−0.0144 (18)	−0.0399 (17)
C15	0.0576 (19)	0.0381 (16)	0.102 (3)	−0.0130 (13)	−0.0099 (17)	−0.0264 (17)
C16	0.0537 (19)	0.0442 (16)	0.073 (2)	−0.0170 (13)	−0.0043 (14)	−0.0122 (15)
C17	0.0473 (16)	0.0415 (14)	0.0591 (17)	−0.0146 (12)	−0.0013 (12)	−0.0211 (13)
C18	0.0543 (19)	0.086 (2)	0.0630 (19)	−0.0170 (16)	−0.0097 (14)	−0.0406 (17)
C19	0.0506 (18)	0.0462 (16)	0.085 (2)	−0.0197 (13)	0.0024 (14)	−0.0335 (15)
C20	0.0489 (18)	0.0526 (18)	0.084 (2)	−0.0073 (14)	−0.0257 (16)	−0.0032 (16)
C21	0.0541 (19)	0.0559 (18)	0.108 (3)	−0.0136 (14)	0.0245 (17)	−0.0487 (18)
N1	0.0331 (12)	0.0320 (11)	0.0558 (13)	−0.0103 (8)	0.0015 (9)	−0.0217 (9)
O1	0.0380 (12)	0.0621 (13)	0.0986 (16)	−0.0187 (9)	0.0098 (10)	−0.0387 (12)

### Geometric parameters (Å, º)

**Table:**

C1—N1	1.464 (3)	C11—H11	0.9300
C1—C6	1.513 (3)	C12—C17	1.375 (4)
C1—C2	1.545 (3)	C12—C13	1.390 (4)
C1—H1	0.9800	C13—C14	1.383 (4)
C2—C19	1.523 (3)	C13—H13	0.9300
C2—C3	1.525 (3)	C14—C15	1.359 (5)
C2—C18	1.534 (4)	C14—H14	0.9300
C3—O1	1.213 (3)	C15—C16	1.372 (4)
C3—C4	1.532 (3)	C15—H15	0.9300
C4—C21	1.523 (4)	C16—C17	1.383 (4)
C4—C20	1.531 (4)	C16—H16	0.9300
C4—C5	1.550 (3)	C17—H17	0.9300
C5—N1	1.460 (3)	C18—H18A	0.9600
C5—C12	1.514 (3)	C18—H18B	0.9600
C5—H5	0.9800	C18—H18C	0.9600
C6—C7	1.378 (4)	C19—H19A	0.9600
C6—C11	1.385 (3)	C19—H19B	0.9600
C7—C8	1.381 (4)	C19—H19C	0.9600
C7—H7	0.9300	C20—H20A	0.9600
C8—C9	1.367 (4)	C20—H20B	0.9600
C8—H8	0.9300	C20—H20C	0.9600
C9—C10	1.367 (5)	C21—H21A	0.9600
C9—H9	0.9300	C21—H21B	0.9600
C10—C11	1.378 (4)	C21—H21C	0.9600
C10—H10	0.9300	N1—H1A	0.94 (3)
			
N1—C1—C6	110.46 (19)	C17—C12—C13	117.6 (2)
N1—C1—C2	109.22 (19)	C17—C12—C5	121.9 (2)
C6—C1—C2	113.51 (18)	C13—C12—C5	120.5 (2)
N1—C1—H1	107.8	C14—C13—C12	120.9 (3)
C6—C1—H1	107.8	C14—C13—H13	119.6
C2—C1—H1	107.8	C12—C13—H13	119.6
C19—C2—C3	109.0 (2)	C15—C14—C13	120.5 (3)
C19—C2—C18	108.4 (2)	C15—C14—H14	119.8
C3—C2—C18	107.4 (2)	C13—C14—H14	119.8
C19—C2—C1	110.9 (2)	C14—C15—C16	119.7 (3)
C3—C2—C1	108.89 (19)	C14—C15—H15	120.2
C18—C2—C1	112.2 (2)	C16—C15—H15	120.2
O1—C3—C2	119.9 (2)	C15—C16—C17	120.0 (3)
O1—C3—C4	118.9 (2)	C15—C16—H16	120.0
C2—C3—C4	121.1 (2)	C17—C16—H16	120.0
C21—C4—C20	108.7 (2)	C12—C17—C16	121.4 (3)
C21—C4—C3	108.7 (2)	C12—C17—H17	119.3
C20—C4—C3	106.0 (2)	C16—C17—H17	119.3
C21—C4—C5	109.6 (2)	C2—C18—H18A	109.5
C20—C4—C5	112.4 (2)	C2—C18—H18B	109.5
C3—C4—C5	111.33 (19)	H18A—C18—H18B	109.5
N1—C5—C12	109.7 (2)	C2—C18—H18C	109.5
N1—C5—C4	110.6 (2)	H18A—C18—H18C	109.5
C12—C5—C4	113.06 (19)	H18B—C18—H18C	109.5
N1—C5—H5	107.7	C2—C19—H19A	109.5
C12—C5—H5	107.7	C2—C19—H19B	109.5
C4—C5—H5	107.7	H19A—C19—H19B	109.5
C7—C6—C11	118.1 (2)	C2—C19—H19C	109.5
C7—C6—C1	121.7 (2)	H19A—C19—H19C	109.5
C11—C6—C1	120.2 (2)	H19B—C19—H19C	109.5
C6—C7—C8	120.9 (3)	C4—C20—H20A	109.5
C6—C7—H7	119.5	C4—C20—H20B	109.5
C8—C7—H7	119.5	H20A—C20—H20B	109.5
C9—C8—C7	120.3 (3)	C4—C20—H20C	109.5
C9—C8—H8	119.8	H20A—C20—H20C	109.5
C7—C8—H8	119.8	H20B—C20—H20C	109.5
C8—C9—C10	119.4 (3)	C4—C21—H21A	109.5
C8—C9—H9	120.3	C4—C21—H21B	109.5
C10—C9—H9	120.3	H21A—C21—H21B	109.5
C9—C10—C11	120.7 (3)	C4—C21—H21C	109.5
C9—C10—H10	119.6	H21A—C21—H21C	109.5
C11—C10—H10	119.6	H21B—C21—H21C	109.5
C10—C11—C6	120.5 (3)	C5—N1—C1	111.10 (19)
C10—C11—H11	119.7	C5—N1—H1A	109.0 (15)
C6—C11—H11	119.7	C1—N1—H1A	111.2 (15)
			
N1—C1—C2—C19	172.5 (2)	N1—C1—C6—C11	−141.9 (2)
C6—C1—C2—C19	−63.7 (3)	C2—C1—C6—C11	95.1 (3)
N1—C1—C2—C3	52.6 (3)	C11—C6—C7—C8	−0.6 (4)
C6—C1—C2—C3	176.3 (2)	C1—C6—C7—C8	179.8 (2)
N1—C1—C2—C18	−66.1 (3)	C6—C7—C8—C9	−0.3 (4)
C6—C1—C2—C18	57.6 (3)	C7—C8—C9—C10	0.8 (5)
C19—C2—C3—O1	25.8 (3)	C8—C9—C10—C11	−0.4 (5)
C18—C2—C3—O1	−91.4 (3)	C9—C10—C11—C6	−0.5 (4)
C1—C2—C3—O1	146.9 (2)	C7—C6—C11—C10	1.0 (4)
C19—C2—C3—C4	−157.6 (2)	C1—C6—C11—C10	−179.4 (2)
C18—C2—C3—C4	85.2 (3)	N1—C5—C12—C17	−37.9 (3)
C1—C2—C3—C4	−36.5 (3)	C4—C5—C12—C17	86.1 (3)
O1—C3—C4—C21	−30.6 (3)	N1—C5—C12—C13	142.3 (2)
C2—C3—C4—C21	152.7 (2)	C4—C5—C12—C13	−93.7 (3)
O1—C3—C4—C20	86.0 (3)	C17—C12—C13—C14	−0.4 (4)
C2—C3—C4—C20	−90.6 (3)	C5—C12—C13—C14	179.4 (2)
O1—C3—C4—C5	−151.4 (2)	C12—C13—C14—C15	−0.6 (4)
C2—C3—C4—C5	31.9 (3)	C13—C14—C15—C16	0.9 (5)
C21—C4—C5—N1	−163.2 (2)	C14—C15—C16—C17	−0.2 (4)
C20—C4—C5—N1	75.8 (3)	C13—C12—C17—C16	1.2 (4)
C3—C4—C5—N1	−42.9 (3)	C5—C12—C17—C16	−178.6 (2)
C21—C4—C5—C12	73.3 (3)	C15—C16—C17—C12	−0.9 (4)
C20—C4—C5—C12	−47.7 (3)	C12—C5—N1—C1	−169.68 (19)
C3—C4—C5—C12	−166.4 (2)	C4—C5—N1—C1	64.9 (2)
N1—C1—C6—C7	37.7 (3)	C6—C1—N1—C5	163.89 (19)
C2—C1—C6—C7	−85.3 (3)	C2—C1—N1—C5	−70.6 (2)

### Hydrogen-bond geometry (Å, º)

**Table:**

*D*—H···*A*	*D*—H	H···*A*	*D*···*A*	*D*—H···*A*
N1—H1*A*···O1^i^	0.94 (3)	2.39 (3)	3.258 (3)	153 (2)

Symmetry code: (i) *x*−1, *y*, *z*.
